# Association of maternal and umbilical cord blood leptin concentrations and abnormal color Doppler indices of umbilical artery with fetal growth restriction

**Published:** 2017-03

**Authors:** Elahe Zareaan, Mitra Heidarpour, Elham Kargarzadeh, Maryam Moshfeghi

**Affiliations:** 1 *Department of Gynecology, School of Medicine, Isfahan University of Medical Sciences, Isfahan, Iran.*; 2 *Department of Pathology, School of Medicine, Isfahan University of Medical Sciences, Isfahan, Iran.*

**Keywords:** Leptin, Fetal growth restriction, Sonography

## Abstract

**Background:**

Fetal growth restriction (FGR) is a condition with heterogeneous pathophysiology which characterized by fetal weight less than the tenth percentile for gestational age. Several factors have impact on maternal, placental and fetal due to growth restriction.

**Objective:**

The aim of this study was to investigate the relationship between levels of leptin in the cord, and serum leptin of mothers also abnormal color Doppler indices of umbilical artery with fetal growth restriction.

**Materials and Methods:**

This is a cross sectional study conducted in Isfahan, Iran, 2015-2016. We recruited 40 women with singleton pregnancies complicated by fetal growth restriction (Group I) and 40 pregnant women with normal fetal growth (Group II) with matched age. Maternal serum and umbilical artery leptin levels were determined with Enzyme-Linked immunosorben method. Also, color Doppler ultrasound of umbilical artery was performed.

**Results:**

Mean maternal and fetal leptin levels were lower in the FGR group compared to the normal group (36.58±(20.99) and 7.42 ±(4.08)vs. 47.32±(22.50) and 30.49±(14.50) respectively). Also, mean fetal leptin level was lower in the group with abnormal color Doppler sonographic indices compared to the normal group (7. 40 ±(4.10)vs 27.06±(15.80), respectively).

**Conclusion:**

This study indicated that maternal and fetal leptin levels are correlated with FGR originating from damaged placental function; also fetal leptin level can indicate changes in color Doppler sonographic indices.

## Introduction

Fetal growth restriction (FGR) is a condition with heterogeneous pathophysiology which characterized by weight less than the tenth percentile for gestational age (1). Several factors with an impact on maternal, placental, and fetal are due to growth restriction. FGR is divided into two symmetrical and asymmetrical types. Asymmetrical type might follow a late pregnancy insult like placental insufficiency from hypertension(2).

Color Doppler ultrasound is a noninvasive technique used to evaluate blood flow. In hypoxic fetal, cerebral vascular impedance is reduced that results in increased blood flow to the brain (3). Fetal vascular dysfunction results in increased umbilical artery blood flow resistance, that is characterized by increased systolic/diastolic (S/D) ratio, Pulsatility Index (PI) or Resistive Index (RI) in the umbilical artery. Following these early changes in fetal with growth restriction, if it being continuous, umbilical artery diastolic blood flow will be change. This changes will be seen in late and progressive FGR (4). Determinants of fetal development are including the provision of necessary material by maternal blood, handling of material through the placenta and fetal growth potential which is controlled by the genome.

However, the precise molecular mechanisms, indicating growth of the fetus,are not fully understood but the role of growth factors such as insulin and insulin-like growth factor has been proved (5, 6). In recent years, other hormones, such as leptin, which are involved in fetal development have been identified (7, 8). Leptin is a 167-amino-acid protein primarily created by adipocytes. Leptin begins to produce by the fetus in the mid-pregnancy period. leptin and its receptors are abundant in placenta amniotic and chorionic. 

Around the 34^th^ week of pregnancy, fetal leptin levels begin to rise and are associated with fetal weight. After birth, maternal and neonatal leptin levels will be decreased (9-14). Despite present data, an association of leptin level with fetal growth and its efficacy during pregnancy to determine pregnancy at risk of FGR is unknown. There are opposite results in this filed for example: Saylon colleague detected no differece between maternal leptin level in the FGR and normal groups (15). Tamura colleague showed fetal leptin level was significantly lower in FGR group but not in mothers like Chiesa colleague (16-17). However, Pighetti *colleague* showed lower fetal leptin level in FGR group but higher maternal leptin level in FGR group like Kyriakakoua and Ferrous (18, 20). 

Our the main goal was to investigate the association of maternal and fetal leptin levels with FGR. Moreover, there is no study to show the relationship between leptin level in complicated pregnancy with Doppler indices changes and severity of FGR.

## Materials and methods

This is a cross sectional study that conducted in Al-zahra and Beheshti hospitals, Isfahan, Iran, from January 2015 until Febuary 2016. We recruited 80 pregnant women (40 women with fetal growth restricted pregnancies and 40 women with normal fetal growth) with matched age. 

The inclusion criteria for participant were, maternal age <40 yr, singleton pregnancy, gestational age >36 wk, non-smoking and substance abuse or teratogenic drug, body mass index (BMI) between 20-27 Kg/m^2^. Exclusion criteria were inability to determine the level of leptin and withdrawal of participants,pregnancy complications, and systemic problems including: kidney disease, anemia, history of diabetes before pregnancy, heart disease, FGR in previous pregnancy, pregnancy with assisted reproductive techniques (ART), and congenital malformations. 

Finally, pregnant women in two groups were formed, Group I: including 40 pregnant women which during prenatal care according to fetal weight below the tenth percentile for gestational age, (which was estimated by ultrasound) has asymmetrical growth restriction. Prenatal care until the termination of pregnancy was performed at the clinic and women underwent to evaluation with color Doppler ultrasound by the umbilical artery. (Measurement of Pulsatility Index (PI), Resistive Index (RI) and systolic/diastolic (S/D) ratio of the umbilical artery as color Doppler indices). Sonography was performed by assistant of obstetrics and gynecology with devices of Mindry company model DC N3 with abdominal probe and 5 megahertz. The biometric indices [(BiParietal Diameter)(BPD), head circumference (HC), abdominal circumference (AC) and femoral length (FL)] according to hadlock formula were measured. 

Finally, based on obstetric indications and according to the findings of Doppler sonography and fetal health assessment for each participants, termination of pregnancy was done in an appropriate manner. It should be noted that all women who had pre-term pregnancy termination were excluded. At the time of hospitalization for pregnancy termination cubital vein blood samples from the mothers were obtained and centrifuged. Also umbilical artery blood samples immediately after birth were prepared and centrifuged. Then samples were stored at -20^o^C and evaluated with Enzyme-linked immunosorben method for measuring the level of leptin according to manufacturer's instructions (LDN company). The laboratory specialists were blinded in this study. 

Group II: included 40 women with normal fetal growth who attended at the clinic for prenatal care and recruited under inclusion criteria. Color Doppler ultrasound in one week interval to termination of pregnancy was performed with the conditions listed above. Finally, at the time of pregnancy termination, maternal and umbilical cord blood samples were prepared and the level of leptin measured in a similar manner.

**Ethical consideration**

The study was approved by Isfahan ethical institutional review board. Written informed consent was obtained from all participants. 

**Statistical analysis**

All data analyses were performed using Statistical Package for the Social Sciences, version 18.0, SPSS Inc, Chicago, Illinois, USA (SPSS). Kolmogorov Smirnov test was used for testing the normality of data. The data presented as mean±SD for continuous variables and number (%) for categorical ones. Mann Whitney U test for comparing non-parametric data and independent sample Student’s T test for comparing normal data were used, too. The Pearson correlation coefficient for determination of correlation between value of leptin with maternal and neonatal weight was applied. Also, ^2^ test was used for testing the association between categorical variables. P<0.05 was considered to be statistically significant. 

## Results

The demographic, clinical, and pregnancy outcome characteristics of participants are presented in table I. The maternal and fetal leptin, were significantly lower in group I, but difference in mean level of fetal leptin in group I compare with group II was very significant. (Table II). According color Doppler sonography,our participants were divided into 2 groups(women with abnormal color Doppler indices (n=33) and normal indices (n=47). Then the maternal serum and fetal leptin levels were compared between these two groups. 

Color Doppler indices was *considered* as abnormal when: increased RI, PI of umbilical artery or the umbilical artery systolic-diastolic (S/D) ratio was above the 95^th^ percentile for gestational age. The mean level of fetal leptin level were shown a significant difference between normal and abnormal color Doppler sonographic groups but mean level of maternal leptin had no significant difference between the two groups (Table III). 

The correlation between maternal serum and fetal leptin with birth weight and BMI in both groups were analyzed separately. In all comparisons, only maternal leptin levels showed significant positive correlation with maternal BMI. Fetal leptin concentrations had no significant correlation with birth weight (R=0.332) and maternal weight (R=0.559) (Table IV).

**Table I:** Demographic, clinical, and pregnancy outcome characteristics in pregnancies complicated by fetal growth restriction (group I) and normal pregnancies (group II

**Characteristics **	**Group I**	**Group II**	**p-value**
Maternal age (yr)	27.28 ± 5.60	28.05 ± 5.30	0.53
Gravidity	2.25 ± 1.20	1.8 0 ± 0.80	0.11
Gestational age at delivery (Wk)	37.10 ± 1.20	37.35 ± 1.20	0.32
BMI (Kg/m^2^)	23.04 ± 2.57	23.18 ± 2.96	0.81
Birth weight (gr)	2321.25 ± 325.48	3219.5 ± 148.2	<0.001

All data presented as mean±SD (Kolmogorov-Smirnov test) (n=40)

BMI: Body mass index

**Table II.** Thecomparison of maternal and fetalleptin level in pregnancies complicated by fetal growth restriction (group I) and normal pregnancies (group II

**Variable**	**Group I **	**Group II **	**p-value**
Maternal serum leptin (ng/ml)	36.56 ± 20.99	47.32 ± 22.50	0.030
Fetal leptin (ng/ml)	7.42 ± 4.08	30.49 ± 14.50	<0.001

Data presented as mean±SD (Kolmogorov-Smirnov test) (n=40)

**Table III:** The maternal and fetal leptin levels in groups with different color Doppler indices

**Color Doppler indices**	**Fetal leptin **	**Maternal Leptin **
Normal (n=47)	27.06 ± 15.80	45.60 ± 22.30
Abnormal (n=33)	7.40 ± 4.10	36.70 ± 21.40
p-value	<0.001	0.07

Data presented as mean±SD (Kruskal-Wallis Test).

**Table IV:** The correlation between maternal and fetal leptin with birth weight and BMI in pregnancies complicated by fetal growth restriction (group I) and normal pregnancies (group II

		**Pearson correlation coefficient**	**p-value**
Maternal leptin and BMI			
	Group I	0.33	0.03*
	Group II	0.37	0.01*
Ma ternal leptin and birth weight			
	Group I	0.07	0.66
	Group II	0.12	0.44
Fetal leptin and birth weight			
	Group I	-0.24	0.12
	Group II	-0.11	0.48
Fetal leptin and BMI			
	Group I	0.087	0.59
	Group II	-2.03	0.21

Pearson correlation

BMI: Body mass index

**Figure 1:**
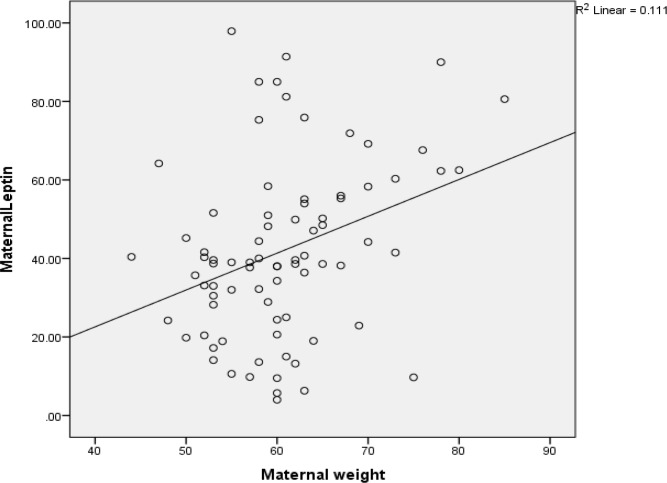
The correlation between maternal weight maternal leptin

**Figure 2:**
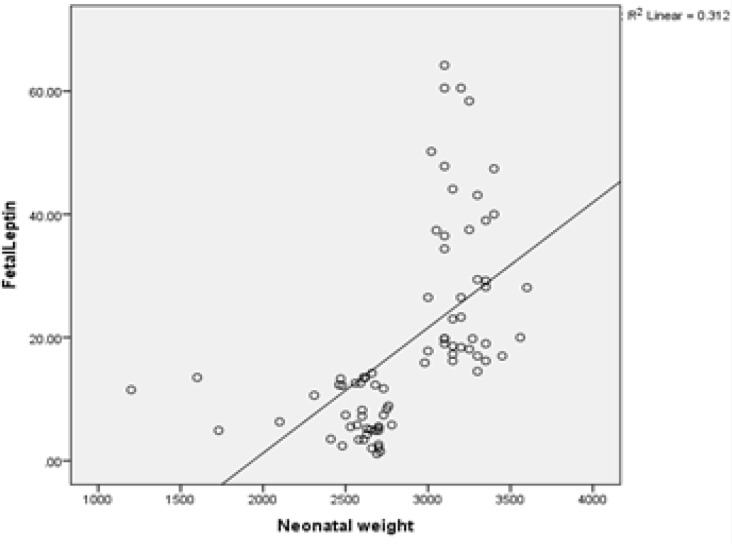
The correlation between neonatal weight fetal leptin

## Discussion

Leptin is a circulating hormone (a 167 amino acid protein) which plays an important role in the regulation of energy balance, placental growth, trophoblast invasion, haemopoiesis and reproduction. Leptin and its receptors are localized in human placental tissue. Mainly the leptin is synthesized in white adipose tissue but the placenta, gastric epithelium, and the brain produce this hormone. There is several evidence for a possible role of leptin in reproduction especially in the fetoplacental physiology:

1- Circulating leptin levels are elevated during pregnancy, reaching a peak during the second trimester.

2- At the end of pregnancy, within 24 hrs. of delivery, maternal plasma leptin levels decline to normal values

3- Leptin is produced by the human placenta

4- Maternal leptin levels are significantly elevated in hydatiform mole, decreasing to normal concentrations after surgery

5- Human first trimester cytotrophoblastic cells secret leptin

6- A high expression of the leptin receptor in human placenta occurs during the third trimester of pregnancy (21).

Most FGR complications have been described as consequences of a lack of maturity and development of organs such as the small intestine, pancreas, spleen, kidneys, and gonads, leading to immediate defects in key biological function.

leptin during the perinatal period is necessary for the development of several peripheral organs, such as the pancreas liver and lung. Moreover, leptin has pronounced effects on body composition, lean mass and body weight and size. As the leptin receptors are widely expressed from an early stage of development, it acts as a key regulatory developmental factor at the cellular and molecular levels (22). Therefore, there are relationship between leptinś function and development. 

According to our result, maternal and especially fetal leptin is associated with FGR and is significantly lower in FGR group compared to normal group. Moreover, disrupted Doppler sonographic indices group had lower leptin level. Saylon *collaegue* study detected no difference between maternal leptin level in FGR and normal groups (15). 

In study by Tamura colleague, no association between maternal leptin and FGR was reported but fetal leptin concentrations were lower and had a significant relationship with birth weight (16). Karamizadeh *et al *in a similar study showed that mean concentration of leptin in cord blood was lower in FGR neonates, but there were no significant difference between maternal leptin (23). As shown in study by Yildiz collaegue, leptin concentrations were significantly lower in FGR group. (n=10; 3.53±1.42 ng/ml, 6.75±1.47 ng/ml, respectively) (24). Chiesa *colleague* showed lower leptin levels in FGR group compare with normal one (17). In opposite, Kyriakakoua *collaegue* reported higher maternal and fetal leptin level in FGR group. Also, Ferrous *collaegue* showed that leptin in FGR group was higher than normal group. (19, 20). Pighetti* colleague* showed lower fetal leptin level in FGR group, but maternal leptin was high in FGR group (18). In any of similar studies the association between leptin and Doppler sonographic indices were not assessed.

## Conclusion

In conclusion, this study showed that maternal and fetal leptin levels correlated with fetal growth restriction originating from damaged placental function, and fetal leptin level can indicate color Doppler indices changes. For further research, we suggest a secondary study (systematic review and Meta-analysis) in association between maternal and fetal leptin with occurrence of FGR in order to verify this evidence.
